# Outcome of autologous stem cell transplantation in patients with favorable-risk acute myeloid leukemia in first remission

**DOI:** 10.1186/s12935-022-02750-4

**Published:** 2022-10-31

**Authors:** Juan Chen, Li Liu, Runzhi Ma, Aiming Pang, Donglin Yang, Xin Chen, Jialin Wei, Yi He, Rongli Zhang, Weihua Zhai, Qiaoling Ma, Erlie Jiang, Mingzhe Han, Jiaxi Zhou, Sizhou Feng

**Affiliations:** 1 State Key Laboratory of Experimental Hematology, Haihe Laboratory of Cell Ecosystem, Institute of Hematology & Blood Diseases Hospital, National Clinical Research Center for Blood Diseases, Chinese Academy of Medical Sciences & Peking Union Medical College, 288 Nanjing Road, Heping District, 300020 Tianjin, China; 2 Department of Hematology, The First Affiliated Hospital of Soochow University, National Clinical Research Center for Hematologic Diseases, Soochow University, 215006 Suzhou, China; 3 Jiangsu Institute of Hematology, The First Affiliated Hospital of Soochow University, Collaborative Innovation Center of Hematology, Key Laboratory of Thrombosis and Hemostasis of Ministry of Health, 215006 Suzhou, China

**Keywords:** Outcome, Autologous stem cell transplantation, Acute myeloid leukemia, Favorable-risk, Remission

## Abstract

**Objective:**

To evaluate the efficacy of autologous hematopoietic stem cell transplantation (auto-HSCT) in patients with favorable-risk acute myeloid leukemia in first remission.

**Method:**

Twenty patients who received auto-HSCT at our center between January 2014 and January 2021 were retrospectively reviewed.

**Results:**

Until last follow-up, three patients in the cohort were dead due to relapse. The estimated 1-year and 5-year overall survival were 95.00% ± 4.87% and 83.82% ± 8.58%, respectively. The estimated 5-year RFS and CIR (cumulative incidence of relapse) were 85.00% ± 7.98% and 15.00% ±7.98%, respectively.

**Conclusion:**

The outcome of auto-HSCT in patients with favorable-risk acute myeloid leukemia in first remission was excellent and auto-HSCT could be an effective treatment for these patients.

## Introduction

According to the ELN risk stratification by genetics, acute myeloid leukemia (AML) can be categorized into favorable-risk, intermediate-risk and adverse-risk groups [1]. For patients in the favorable-risk group, chemotherapy and hematopoietic stem cell transplantation (HSCT) (both auto and allo) are post-remission treatment choice. Consolidation chemotherapy including high dose cytarabine is recommended for favorable-risk AML in first remission by the NCCN (National Comprehensive Cancer Network) guideline [2]. However, a considerable proportion of these patients will experience disease relapse after chemotherapy [3, 4]. Allogeneic hematopoietic stem cell transplantation (allo-HSCT) can significantly reduce the relapse rate due to the graft-versus-leukemia (GVL) effect. However, the lack of HLA matched sibling donor and high nonrelapse mortality (NRM) limit its application. Autologous hematopoietic stem cell transplantation (auto-HSCT) may offer lower transplantation-related mortality (TRM) compared with allogeneic HSCT and lower relapse rate comparing to chemotherapy [5–9]. Therefore, auto-HSCT is a promising treatment for patients with favorable-risk AML as it may improve survival with relatively low TRM. In this study, we aimed to assess the efficacy of autologous HSCT in patients with favorable-risk acute myeloid leukemia in first remission.

## Patients and methods

### Patients

We retrospectively analyzed the data of 20 consecutive patients with acute myeloid leukemia in first remission who received autologous HSCT at Institute of Hematology, Chinese Academy of Medical Science from January 2014 and January 2021. The patients were categorized as favorable-risk according to the ELN risk stratification by genetics for AML [1].

The final date of follow-up was January 2022 for patients without events.

All patients provided written informed consent for this protocol. For patients younger than 18 years old in the cohort, the consent was carried out by their parents. This study was approved by the Ethics Review Committee of the Institute of Hematology, Chinese Academy of Medical Science & Peking Union Medical College and was in compliance with the Declaration of Helsinki.

### Cytogenetic and molecular analysis

The cytogenetic analysis was conducted at diagnosis by karyotye and/or fluorescence in situ hybridization. Molecular analysis including gene mutation and fusion gene screening was performed by next generation sequencing at diagnosis. Minimal residual disease (MRD) for RUNX1-RUNX1T1, CBFB-MYH11 and NPM1 was determined by real-time quantitative polymerase chain reaction analysis, while for biallelic mutated CEBPA, it was determined by Sanger sequencing.

### Conditioning regime

All patients undergoing auto-HSCT received conditioning based on Bu (busulfan): 0.8 mg/kg q6h*3d or TBI (total body irradiation): 3.3 Gy/d*3d, Flu (fludarabine): 30 mg/m^2^*3d or Cla (Cladribine): 5 mg/m^2^*3d, Ara-c (cytarabine): 1-2 g/m^2^*3d, and 14 patients received Cy (cyclophosphamide): 40 mg/kg*2d in addition.

### Transplantation details

Transplantation associated details were shown in Table 1. Seventeen patients received stem cells from donor peripheral blood (PB), 2 patients from PB plus bone marrow (BM), and 1 patient from BM because of poor mobilization of PB stem cells. The median dose of infused MNC and CD34^+^ cells were 10.90*10^8^/kg (range from 2.33 to 30.55*10^8^/kg) and 3.28 *10^6^/kg (range from 1.16 to 3.23*10^6^/kg), respectively.

**Table 1:** Transplantation details and outcomes

Genetic abnormality	Pre-conditioning	Graft source	MNC	CD34^+^ cells	ANC engraftment	PLT engraftment	Follow-up	Relapse	Death
t(8;21)(q22;q22.1); RUNX1-RUNX1T1 (N = 3)	Bu + Ara-C	PB	6.94	1.63	14	154	2840	No	No
	Bu + Cy + Flu + Ara-C	PB	10.5	1.95	12	17	2521	No	No
	TBI + Cy + Cla + Ara-C	PB	10.9	1.64	34	60	1346	No	No
inv(16)(p13.1q22) or t(16;16)(p13.1;q22); CBFB-MYH11 (N = 3)	Bu + Flu + Ara-C	PB	10.44	2.13	12	14	2692	No	No
	TBI + Cy + Flu + Ara-C	PB + BM	4.14	2.08	43	50	2545	No	No
	BU + Cla + Ara-C	PB	11.41	1.52	11	35	345	No	No
Biallelic mutated CEBPA (N = 5)	Bu + Cy + Cla + Ara-C	PB	12.89	1.87	13	23	1033	No	No
	Bu + Cy + Flu + Ara-C	PB	6.09	2.44	13	33	2097	No	No
	Bu + Cy + Flu + Ara-C	BM	2.33	1.61	40	60	2658	No	No
	BU + Cy + Cla + Ara-C	PB	10.63	2.43	12	15	1362	No	No
	Bu + Cla + Ara-C	PB	17.86	1.76	14	28	911	No	No
Mutated NPM1 without FLT3-ITD or with FLT3-ITD^low^ (N = 9)	Bu + Cy + Flu + Ara-C	PB	30.55	2.80	12	282	463	Yes	Yes
	Bu + Cy + Cla + IDA	PB	8.35	1.70	13	17	1821	No	No
	Bu + Cy + Flu + Ara-C	PB	11.31	2.40	12	16	2210	No	No
	Bu + Cla + Ara-C	PB	8.33	2.67	11	17	407	No	No
	Bu + Cy + Flu + Ara-C	PB	11.56	1.16	17	70	2238	No	No
	Bu + Cy + Flu + Ara-C	PB + BM	17.54	2.13	15	43	94	Yes	Yes
	Bu + Cy + Cla + Ara-C	PB	2.88	3.23	11	14	408	Yes	Yes
	Bu + Cla + Ara-C	PB	20.97	2.10	16	39	985	No	No
	BU + Cy + Cla + Ara-C	PB	11.38	2.50	14	39	1720	No	No

### Criteria of outcomes

Engraftment was defined as ANC (absolute neutrophil counts) ≥ 0.5 × 10^9^/L for three consecutive days and platelet counts ≥ 20 × 10^9^/L without transfusion for 7 consecutive days.

Overall survival (OS) was defined from HSCT to death of any cause or last follow-up. Relapse free survival (RFS) was calculated as the time from transplantation to relapse or the end of follow-up. Relapse was defined as at least 5% leukemia blasts in a BM smear, or extramedullary leukemia.

### Statistical analysis

The data were analyzed by the software GraphPad Prism 8 and IBM SPSS statistics 25. The Kaplan-Meier method was used to estimate the cumulative survival/incidence. The descriptive statistics for continuous variables was used to compare incidence in univariate analysis. The Kaplan-Meier method was used to estimate the cumulative survival/incidence.

## Results

### Characteristics of patients

The 20 patients were favorable-risk AML categorized by genetics, of which 3 patients were t(8;21)(q22;q22.1); RUNX1-RUNX1T1, 3 patients were inv(16)(p13.1q22) or t(16;16)(p13.1;q22); CBFB-MYH11, 5 patients were biallelic mutated CEBPA, and 9 patients were normal karyotype with mutated NPM1 without FLT3-ITD. The baseline characteristics of patients in the four groups were listed in Table 2. Median leukocyte counts at diagnosis were 26.68*10^9^/L (range from 1.11 to 190.37*10^9^/L). Three patients were with additional cytogenetic abnormities, 2 with -Y and 1 with + 22. Seven patients had additional molecular abnormality, 3 with FLT3-TKD, 1 with NF1 and RELN mutation, 1 with c-KIT mutation, 1 with TET2 mutation, and 1 with IDH1 mutation. All patients had complete remission after 1 or 2 induction courses of chemotherapy, and received a median of 3.5 (range from 1 to 6) additional consolidation courses of chemotherapy including medium-dose cytarabine before transplantation. MRD was negative for all patients before auto-HSCT.

**Table 2:** Baseline Characteristics of Patients

Genetic abnormality	Age (years)	Gender	Leukocyte at diagnosis	Months before transplantation	Additional cytogenetics	Additional molecular abnormality	Chemotherapy before CR	Consolidation course	MRD
t(8;21)(q22;q22.1); RUNX1-RUNX1T1 (N = 3)	51	Male	21.83	11	-Y	/	1	4	Negative
	45	Male	19.42	8	-Y	/	1	3	Negative
	16	Male	3.77	8	/	/	1	3	Negative
inv(16)(p13.1q22) or t(16;16)(p13.1;q22); CBFB-MYH11 (N = 3)	19	Male	86.28	12	/	FLT3-TKD	1	6	Negative
	25	Male	190.37	8	+ 22	FLT3-TKD	1	4	Negative
	42	Female	1.11	6	/	NF1-RELN	1	1	Negative
Biallelic mutated CEBPA (N = 5)	39	Male	16.3	7	/	/	2	3	Negative
	34	Male	13.5	8	/	/	1	4	Negative
	41	Male	23.39	9	/	C-KIT	1	4	Negative
	35	Male	27.10	7	/	/	1	3	Negative
	17	Female	40.53	6	/	/	1	2	Negative
Mutated NPM1 without FLT3-ITD (N = 9)	59	Female	26.25	11	/	/	1	4	Negative
	26	Female	35.69	11	/	/	1	5	Negative
	56	Male	30.00	8	/	/	1	4	Negative
	33	Female	25.97	9	/	/	1	4	Negative
	20	Male	44.70	9	/	/	2	3	Negative
	56	Female	124.00	10	/	/	1	5	Negative
	33	Female	56.87	5	/	FLT3-TKD	1	2	Negative
	30	Male	5.77	8	/	TET2	1	3	Negative
	41	Male	101.80	7	/	IDH1	1	2	Negative

### Engraftment

All patients had ANC engraftment and the median time of engraftment was 13 days (range from 11 to 43 days). For platelet, the median time of engraftment was 33 days (range from 14 to 282 days) and 18 patients (90%) had platelet engraftment in 100 days post transplantation.

### Deaths and survival

**Fig. 1:**
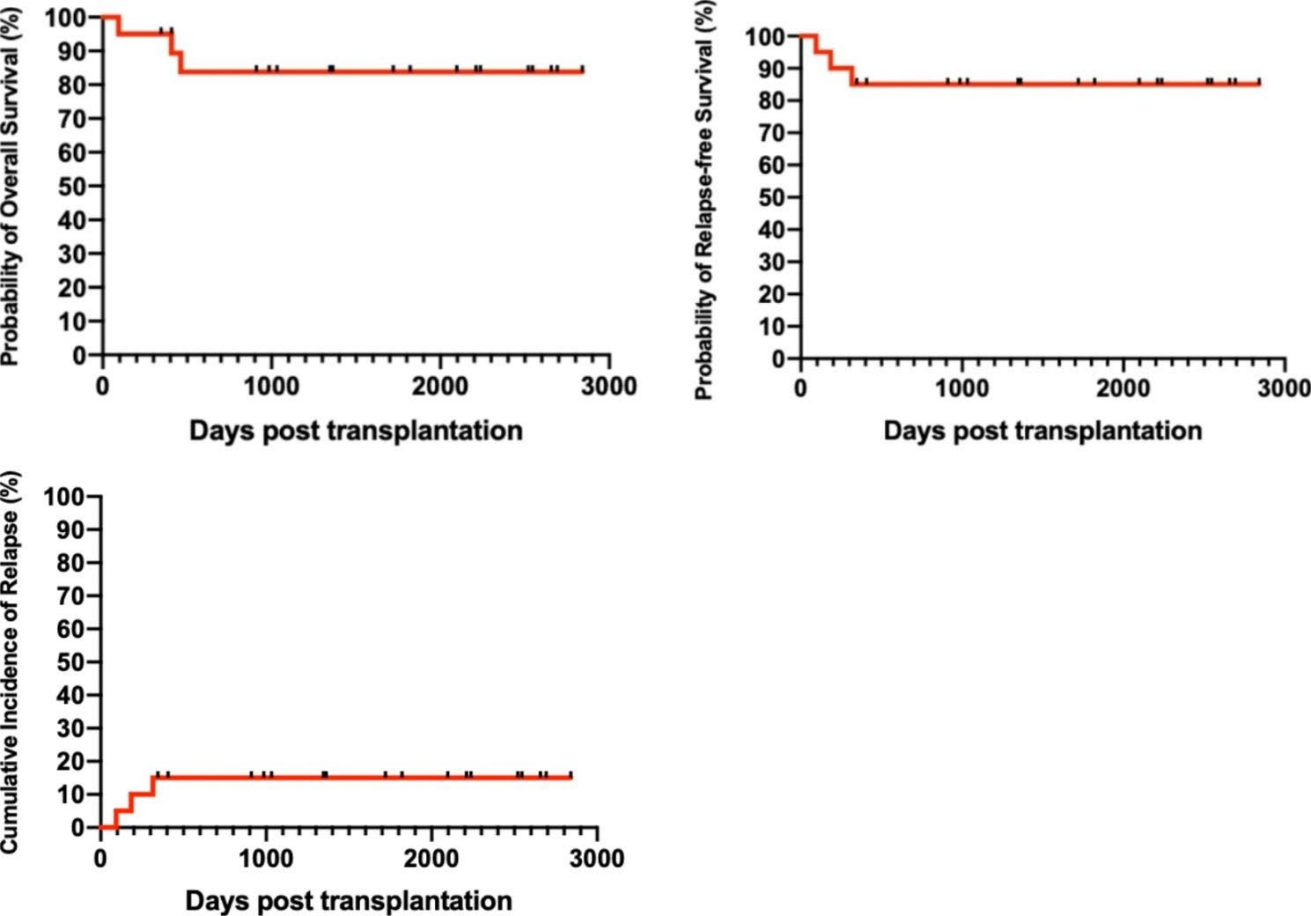
Survival and relapse analysis of patients. (A) Overall survival, (B) relapse-free survival, and (C) cumulative incidence of relapse

The median follow up of the patients was 1362 days (range from 94 to 2840 days). Of all the patients, three patients received cytokine induced killer (CIK) cell infusion after transplantation, three patients received interleukin-2 therapy and one patient received azacitidine treatment for maintaining therapy. Until the last follow up, three patients died and they all died of relapse. Time to relapse from HSCT was 94 days, 184 days and 315 days, respectively. The relapsed patients did not receive maintaining therapy after transplantation. Median OS for the nonrelapse patients were 1821 days (range from 345 to 2840 days). The estimated 1-year and 5-year OS were 95.00% ± 4.87% and 83.82% ± 8.58%, respectively. The estimated 5-year RFS and CIR (cumulative incidence of relapse) were 85.00% ± 7.98% and 15.00% ±7.98%, respectively (Fig. 1).

## Discussion

AML is a kind of heterogeneous diseases and currently stratified by karyotype and molecular characteristics. Patients with t(8;21)(q22;q22.1); RUNX1-RUNX1T1, inv(16)(p13.1q22) or t(16;16)(p13.1;q22); CBFB-MYH11, biallelic mutated CEBPA or mutated NPM1 without FLT3-ITD or with FLT3-ITD^low^ were thought to be favorable-risk with good prognosis [1]. Although these patients can achieve complete remission easily after induction chemotherapy, high relapse rate was observed for patients receiving consolidation chemotherapy alone [3]. Allo-HSCT can reduce relapse significantly, but it is a little aggressive regarding favorable-risk AML in first remission and usually not considered due to high nonrelapse mortality. The role of auto-HSCT for AML is controversial since it lacks GVL effect which may be associated with increased relapse risk [10–14]. Given this context, we aimed to evaluate the efficacy of auto-HSCT for these patients.

Several previous studies [4, 15] has focused on auto-HSCT in patients with core binding factor (CBF) AML, including t(8;21)(q22;q22.1); RUNX1-RUNX1T1, inv(16)(p13.1q22) and t(16;16)(p13.1;q22); CBFB-MYH11, which accounts for 10–15% of all AML patients [16–18]. The estimated 5-year OS and CIR for these patients were 84-89% and 16-17.5%, respectively. Other studies [5, 7] evaluated auto-HSCT for patients with favorable-risk AML also showed good efficacy. Moreover, comparing to allo-HSCT, the efficacy was identical. A recent study comparing the efficacy of auto-HSCT and haplo-HSCT (haploidentical HSCT) for favorable-risk AML patients found that there was no significant difference in 3-year OS between the two groups ( 88.3%±5.2% vs. 93.1%±4.7%, P = 0.318)^8^. Another study from the EBMT (European Cooperative Group for Blood and Marrow Transplantation) also showed similar outcomes after auto-HSCT or allo-HSCT in first remission of AML carrying inversion 16 or t(8;21) [18].

In the current study, we retrospectively analyzed the data of 20 patients with favorable-risk AML who underwent auto-HSCT in first remission and identified excellent efficacy. With a median follow up of 1362 days (range from 94 to 2840 days), 17 (85.00%) patients survived without relapse. The estimated 1-year and 5-year OS were 95.00% ± 4.87% and 83.82% ± 8.58%, respectively, which corresponded well with other studies. The estimated 5-year RFS and CIR were also similar to previous studies [4, 7, 8, 15]. MRD status before auto-HSCT was suggested as an independent prognostic factor for both OS and RFS [7, 8]. Three patients experienced relapse (all in one-year) and responded poorly to subsequent chemotherapy and then died unfortunately. This result was consistent with previous study [15], indicating that relapse was the leading cause of death for auto-HSCT and should be paid more attention with may be more frequent MRD assessments. Moreover, in our study, the three relapsed patients had relatively older age [56 years old (range from 33 to 59 years old) vs. 34 years old (range from 16 to 56 years old), P = 0.053] and higher leukocyte counts [56.87*10^9^/L (range from 26.25 to 124.00 *10^9^/L) vs. 25.97*10^9^/L (range from 1.11 to 190.37 *10^9^/L), P = 0.348] at diagnosis comparing to other patients. These two factors (especially old age) were prognostic factors for auto-HSCT identified by many studies [5, 19–21] and may be associated with relapse, but pending further confirmation.

In conclusion, auto-HSCT could achieve excellent outcome with low relapse rate for young favorable-risk AML patients in first remission with MRD negativity. The present study provided evidence in clinical application of auto-HSCT for these patients. However, due to the retrospective origin and small sample size, future prospective, large-scaled clinical trials are needed to investigate and confirm the efficacy and more efforts should be made to furtherly reduce relapse.

## Data Availability

The data and materials can be obtained from the first author and corresponding author.
